# Needlestick and sharps injuries among health care workers at public tertiary hospitals in an urban community in Mongolia

**DOI:** 10.1186/1756-0500-4-184

**Published:** 2011-06-14

**Authors:** Mayo Kakizaki, Nayu Ikeda, Moazzam Ali, Budbazar Enkhtuya, Muugolog Tsolmon, Kenji Shibuya, Chushi Kuroiwa

**Affiliations:** 1 NTT Communications Corporation, Tokyo, Japan; 2 Department of Global Health Policy, Graduate School of Medicine, the University of Tokyo, 7-3-1 Hongo, Bunkyo-ku, Tokyo 113-0033, Japan; 3 Department of Reproductive Health and Research, World Health Organization, Geneva, Switzerland; 4 Department of Immunization, National Center of Communicable Diseases, Ministry of Health, Ulaanbaatar, Mongolia; 5 Research and Development Division, National Center of Communicable Diseases, Ministry of Health, Ulaanbaatar, Mongolia; 6 Yotsukaido Tokushukai Medical Center, Chiba, Japan

**Keywords:** needlestick and sharps injuries, infection control, blood-borne pathogens, universal precautions, Mongolia

## Abstract

**Background:**

Needlestick and sharps injuries (NSSIs) are one of the major risk factors for blood-borne infections at healthcare facilities. This study examines the current situation of NSSIs among health care workers at public tertiary hospitals in an urban community in Mongolia and explores strategies for the prevention of these injuries.

**Findings:**

A survey of 621 health care workers was undertaken in two public tertiary hospitals in Ulaanbaatar, Mongolia, in July 2006. A semi-structured and self-administered questionnaire was distributed to study injection practices and the occurrence of NSSIs. A multiple logistic regression analysis was performed to investigate factors associated with experiencing NSSIs. Among the 435 healthcare workers who returned a completed questionnaire, the incidence of NSSIs during the previous 3 months was 38.4%. Health care workers were more likely to report NSSIs if they worked longer than 35 hours per week (odds ratio, OR: 2.47; 95% confidence interval, CI: 1.31-4.66) and administered more than 10 injections per day (OR: 4.76; 95% CI: 1.97-11.49). The likelihood of self-reporting NSSIs significantly decreased if health care workers adhered to universal precautions (OR: 0.34; 95% CI: 0.17-0.68).

**Conclusions:**

NSSIs are a common public health problem at public tertiary hospitals in Mongolia. The promotion of adequate working conditions, elimination of excessive injection use, and adherence to universal precautions will be important for the future control of potential infections with blood-borne pathogens due to occupational exposures to sharps in this setting.

## Background

Percutaneous exposures to blood and body fluids through contaminated needlesticks and sharps are an important occupational hazard for morbidity and mortality from infections with blood-borne pathogens among health care workers[1,2]. Among the 35 million health care workers worldwide, three million experience needlestick and sharps injuries (NSSIs) every year,[3] with a high incidence of these injuries being reported from health care facilities in a number of countries that vary in terms of their level of economic development[4-12]. NSSIs pose a considerable risk for the transmission of more than 20 kinds of blood-borne pathogens, such as hepatitis B virus (HBV), hepatitis C virus (HCV), and human immunodeficiency virus (HIV)[2]. The World Health Organization has estimated that exposure to sharps in the workplace accounts for 40% of infections with HBV and HCV and 2-3% of HIV infections among health care workers[3].

In order to develop effective policy measures for reducing the risk of NSSIs, it is essential to understand what contributes to the incidence of these injuries in clinical practice. Previous studies have revealed that NSSIs are more likely to occur among health care workers who are female,[13] young,[4,8,9,13] white and non-Hispanic,[13] anesthesiology technicians [4], gynecologists/obstetricians and surgeons,[10] working mixed shifts,[8,14] working long hours,[9,11,14] working in surgical or intensive care units,[9] have less working experience,[9] are recapping needles,[11] are not using protective gloves when handling needles,[11] and are not involved in health and safety issues or not appropriately trained in procedures for risk control[6,11]. Policymakers and hospital administrators need to reflect on these factors in the formulation and implementation of health promotion strategies for the prevention of NSSIs.

Prevention of NSSIs is one of the major public health issues in Mongolia, where safer sharps devices, or devices with built-in safety features or mechanism to effectively reduce the risk of the injuries are not widely available. An earlier study on injection practices in this country demonstrated that more than half of the health staff who had administered injections reported one or more NSSIs during the past 12 months and recapping needles using the two-handed method, whereas none of them reported completion of three doses of the hepatitis B vaccine[15]. Although this previous study was based on only a small sample of 28 injection providers, these findings nevertheless suggest that an in-depth analysis is necessary to investigate which factors account for the occurrence of NSSIs in Mongolia.

The present study aims to examine the current situation of infection control through the management and prevention of NSSIs among health care workers at tertiary hospitals in the capital of Mongolia. Circumstances and factors surrounding the occurrence of NSSIs are explored to provide evidence for establishing effective occupational safety standards and precautions regarding the handling of blood-related products at healthcare facilities in this country.

## Methods

### Data

A survey was conducted in July 2006 of all health care workers at two public tertiary hospitals in Ulaanbaatar, Mongolia, to study their practice of handling needlesticks and sharps and the management of NSSIs. Two out of thirteen public tertiary hospitals in the capital city were selected for this study: the National Center for Communicable Diseases (NCCD) Hospital and P.N. Shastin Central Hospital (commonly called Shastin Hospital). These hospitals were recommended as survey sites by the Ministry of Health in Mongolia, because they were the largest of the 13 tertiary hospitals. The NCCD Hospital has 400 beds and accepts 90,000 outpatients every year, while Shastin Hospital has 510 beds and receives 150,000-200,000 outpatients annually[16]. NCCD specializes in communicable diseases and has units specializing in hemodialysis, nephrology, hematology, urology and endocrinology[16]. Shastin Hospital has a surgical department and the only cardiac care center in Mongolia as well as units specializing in neurology, pulmonology, ophthalmology, and nephrology[16].

A researcher visited each hospital on one day during the survey period to distribute and collect a self-administered questionnaire. All 621 health care workers at the two hospitals were eligible to participate in this study, including physicians, surgeons, nurses, laboratory assistants and ancillary staff. They were considered to be at risk of NSSIs as a result of handling sharps contaminated with blood, such as needles, scalpels, and lancets. Health care workers were excluded from the survey if they were unable to be present at a survey site at the appointed time because they had to attend an emergency.

We developed a semi-structured questionnaire consisting of questions inquiring about five topics: 1) demographic characteristics and working conditions; 2) daily injection practices; 3) education and training at the hospital; 4) the occurrence of NSSIs in the previous three months; and 5) respondents' knowledge, attitudes and practice concerning the prevention and management of NSSIs. Reporting on the occurrence of NSSI was limited to the previous 3 months in order to minimize the possibility of recall bias.

The questionnaire was based on three published sources. The first was the "Injection Practices: Rapid Assessment and Response Guide" of the World Health Organization[17]. This guideline provides a questionnaire to assess injection practices at national and regional levels. The questionnaire was pilot tested in more than twenty countries and was posted on the Internet forum site of the Safe Injection Global Network from October 2000 to August 2002 to elicit comments from the public audience. The second source was the "Needlestick and Sharps Object Injury Report" prepared by the Exposure Prevention Information Network[18]. This report presented a map of the human body divided into 62 parts, so that participants could identify injured parts of the body. The third source was the universal precautions of the Centers for Disease Control and Prevention for preventing the transmission of HBV, HIV and other blood-borne pathogens among health care workers. Universal precautions consider all patients to be potential carriers of blood-borne viruses[19].

The questionnaire was initially constructed in English. It was subsequently translated into the Mongolian language and was then back-translated into English by a Mongolian researcher who was fluent in both languages.

The research protocol and questionnaire was approved by the Institutional Ethical Review Board at the University of Tokyo and the Ministry of Health, Mongolia. Written informed consent was obtained from all participants.

### Statistical analysis

The incidence of NSSIs in the preceding three months was estimated. A respondent was considered to have had an experience of NSSIs if they self-reported that they had one or more NSSIs during the three months prior to the survey. A multiple logistic regression analysis was performed to explore factors relating to the occurrence of NSSIs. Explanatory variables included respondents' gender, job categories (nurses, medical doctors, surgeons, housekeepers, and others), the length of service at the present hospital (≤ 10, 11-20, and > 20 years), working hours per week (≤ 35 and > 35 hours), the number of injections administered per day (zero, 1-10, and > 10 injections), the practice of recapping needles ("not recapping," "recapping with the one-handed method," and "recapping with the two-handed method"), and a series of binary variables indicating 1 for "working night shifts," "receiving education on injury prevention at hospital," "knowing hospital policies on injury prevention," "following universal precautions," "being supplied with sufficient safety wear," and "working at a surgical department." P-values under 0.05 were considered to indicate statistical significance. All analyses were conducted with Stata/SE version 10.1 for Windows (StataCorp, Texas, USA).

## Results

Of 621 eligible health care workers at the two target hospitals, 438 participated in the survey (response rate, 70.5%). After three cases were excluded for providing incomplete information, a sample of 435 was obtained for this analysis: 213 (49.0%) from the NCCD and 222 (51.0%) from Shastin Hospital. Table 1 summarizes the basic characteristics and working environment of the respondents by hospital. A majority of the respondents were female and working as nurses.

**Table 1:** Basic characteristics and working environment of study participants by hospital

Variable	NCCD		Shastin		Total	
Age in years, mean (SD)	40.0	(8.9)	36.2	(8.7)	38.0	(9.0)
Sex						
Female	194	(92.4)	190	(88.0)	384	(90.1)
Male	16	(7.6)	26	(12.0)	42	(9.9)
Job category						
Nurse	134	(66.7)	123	(59.7)	257	(63.1)
Medical doctor	29	(14.4)	12	(5.8)	41	(10.1)
Surgeon	4	(2.0)	19	(9.2)	23	(5.7)
Ancillary staff	15	(7.5)	16	(7.8)	31	(7.6)
Others	19	(9.5)	36	(17.5)	55	(13.5)
Working in a surgical unit	9	(5.3)	27	(14.2)	36	(10.0)
Length of service in years, median (IQR)	20	(9-26)	12	(6-20)	16	(7-23)
Working hours per week, median (range)	35	(6-56)	40	(5-80)	35	(5-80)
Frequency of night shifts per week						
None	91	(43.8)	95	(45.5)	186	(44.6)
Once	22	(10.6)	10	(4.8)	32	(7.7)
Twice	66	(31.7)	49	(23.4)	115	(27.6)
≥ 3 times	29	(13.9)	55	(26.3)	84	(20.1)
Number of injections per day, median (IQR)	2	(0-14)	1	(0-15)	2	(0-15)
Attendance at educational sessions						
Yes	96	(48.0)	87	(43.9)	183	(46.0)
No	104	(52.0)	111	(56.1)	215	(54.0)
Knowing hospital policies						
Yes	142	(74.7)	161	(86.1)	303	(80.4)
No	48	(25.3)	26	(13.9)	74	(19.6)
Following universal precautions						
Yes	57	(33.3)	60	(34.3)	117	(33.8)
No	114	(66.7)	115	(65.7)	229	(66.2)
Recapping needles						
Not recapping	161	(83.4)	120	(63.5)	281	(73.6)
Recapping with one-handed method	18	(9.3)	40	(26.2)	58	(15.2)
Recapping with two-handed method	14	(7.3)	29	(15.3)	43	(11.3)
Being supplied with adequate safety wear						
Yes	157	(86.7)	157	(84.9)	314	(85.8)
No	24	(13.3)	28	(15.1)	52	(14.2)

IQR, inter-quartile range; NCCD, National Center for Communicable Diseases; SD, standard deviation.

Values are numbers (percentages) unless stated otherwise.

Denominators of the percentages vary by the number of missing values.

The incidence of NSSIs during the three months prior to the survey was 38.4% (167/435). The frequency of incidence was once for 14.7% (64/435), twice for 11.0% (48/435), and three times or more for 12.6% (55/435). The incidence of NSSIs differed significantly between the hospitals: 29.1% (62/213) at NCCD and 47.3% (105/222) at Shastin Hospital (P < 0.001). One fifth of the respondents at Shastin Hospital (45/222) reported having experienced three or more NSSIs in the preceding three months, while this figure was significantly lower at NCCD, being only 4.7% (10/213, P < 0.001).

Regarding the injection practice of survey participants, the median number of injections they administered per day was 2 (interquartile range: 0-15). A majority of the respondents (92.1%, 350/380) reported that they had not reused needles after sterilization. Almost three quarters of them (73.6%, 281/382) reported that they did not recap a needle, while 15.2% (58/382) and 11.3% (43/382) reported that they recapped a needle using the one-handed method and the two-handed method, respectively.

As regards their knowledge, attitudes, and practice of NSSIs, 46.0% of 398 respondents had attended educational programs on injury prevention at their hospital in the preceding year; 49.2% (146/297) had ever taken training sessions on blood collection; 80.4% (303/377) knew hospital policies on the prevention of NSSIs; and 85.8% (314/366) had a sufficient supply of safety wear. However, only 33.8% of 346 respondents followed universal precautions and treated all patients as if they were a carrier of potential blood-borne viruses. The most frequent reason for not adhering to universal precautions was that they did not think that universal precautions were important. In addition, of 167 respondents who reported at least one NSSI in the previous three months, 64.1% answered that they had sought medical assistance after the occurrence of an injury. The most common reason for not seeking medical assistance after the occurrence of an injury was that they did not know where to seek medical assistance.

Table 2 shows the adjusted odds ratios for the factors related to the occurrence of NSSIs during the three months prior to the survey. The regression analysis was performed on 234 cases that had valid values on all of the variables included in the model. After controlling for confounding factors, NSSIs were almost 2.5 times more likely to occur among health care workers who worked longer than 35 hours per week (P = 0.005). Compared to health care workers who didn't administer any injections, the likelihood of experiencing NSSIs was about five times higher among those who gave more than 10 injections per day (P = 0.001). Moreover, health care workers who followed universal precautions were 66% less likely to have NSSIs than were those who did not adhere to these recommendations (P = 0.002).

**Table 2:** Adjusted odds ratios for experiencing needlestick and sharps injuries in the previous three months (n = 234)

Explanatory variables	Odds ratio	95% confidence interval
Sex		
Female	1.00	Reference
Male	1.33	0.34-5.26
Job category		
Nurses	1.00	Reference
Medical doctor	1.15	0.35-3.77
Surgeon	3.50	0.38-32.33
Ancillary staff	2.52	0.58-10.98
Other	1.24	0.43-3.58
Working in a surgical unit	1.48	0.52-4.25
Length of service (years)		
≤ 10	1.00	Reference
11-20	0.60	0.29-1.26
> 20	0.56	0.27-1.17
Working hours per week		
≤ 35	1.00	Reference
> 35	2.47	1.31-4.66
Having night shifts	1.15	0.60-2.21
Number of injections per day		
Zero	1.00	Reference
1-10	1.80	0.78-4.15
> 10	4.76	1.97-11.49
Attendance at educational sessions	1.68	0.89-3.17
Not knowing hospital policies	1.24	0.84-1.81
Following universal precautions	0.34	0.17-0.68
Recapping needles		
Not recapping	1.00	Reference
Recapping with one-handed method	1.36	0.63-2.94
Recapping with two-handed method	1.12	0.38-3.30
Being supplied with adequate safety wear	1.22	0.80-1.87

## Discussion

More than one third of health care workers at the two largest public tertiary hospitals in Ulaanbaatar, Mongolia, were exposed to the risk of infection with blood-borne pathogens through their contact with needlesticks and sharps. This study revealed for the first time that, in an urban area in Mongolia, NSSIs were more likely to be reported from health care workers who handled sharps at tertiary hospitals if they worked long hours, administered a high number of injections each day, or did not adhere to universal precautions.

The incidence of NSSIs in the present study was substantially lower than the figure from an earlier study in Mongolia (57%)[15]. It cannot be ascertained from the current study whether the incidence of NSSIs has decreased or if the difference is attributable to the superior performance of the study hospitals. A part of the difference, however, may be related to the different recall periods between the two studies: three months in the current study and twelve months in the previous study. The present study adopted three months as a recall period in order to minimize recall errors, although twelve months has been a popular timeframe in past studies.

Long working hours were positively associated with the occurrence of NSSIs, which is consistent with past studies[9,11,14]. This finding confirms a need for keeping adequate working hours to reduce the risk of these injuries and infection with blood-borne pathogens in Mongolia. This may be particularly relevant for Shastin Hospital, where half of the health care workers were working for 40 hours or more per week at the time of the survey.

The number of injections given per day had a positive association with the occurrence of NSSIs, which is also consistent with previous studies[20]. The probability of unsafe injections and consequent NSSIs may increase if health care workers have to give an excessive number of injections. The number of injections administered per day should be regularly monitored to avoid unnecessary injections and a shift toward alternative treatments such as oral medicines should be encouraged. This finding may be of particular importance for Mongolia, where the preference for injections is relatively high as a result of the legacy of the former socialist system[15].

This study showed that adherence to the universal precautions recommendations was another important factor for the prevention of NSSIs in Mongolia, a finding which is in accord with past studies in other countries[21,22]. One third of the health care workers did not follow universal precautions at their tertiary hospitals in Ulaanbaatar. Unsafe injection practices, such as reusing and recapping needles after giving injections, are still observed among them. Given that safer sharps devices or devices with a built-in safety feature is not yet widely available in Mongolia, strengthening education and training systems is thus essential. This should help ensure that health care workers attend seminars designed to enhance their awareness of the standard precautions and protocols, knowledge of which seems to be still far from adequate in the country.

This study has demonstrated that the inadequate reporting of NSSIs to medical staff was common among health care workers in Mongolia--as many as one third of the survey participants did not seek medical treatment after an injury incident--as has been reported from previous studies in other countries[6,7,21]. As post-exposure prophylaxis has been shown to be effective after these injuries,[20] a system should be introduced to ensure that all health care workers know about where to seek medical treatment after the occurrence of NSSIs.

This study had several limitations that need to be considered when interpreting the results. First, the estimated incidence of NSSIs and their associated factors may be subject to reporting errors, because all the information came from the self-reports of the survey participants themselves. In particular, social desirability bias may have been present in the form of the underreporting of NSSIs in the survey. The introduction of a system for the computerized collection of information on work records and NSSIs would facilitate the production of accurate data for monitoring the occurrence of injuries and the management of sharps and medical waste. Second, as briefly mentioned above, the present study limited the NSSI's recall period to three months prior to the survey in order to minimize recall bias among the respondents. Hence, the estimated incidence rates cannot be directly compared with those of earlier studies that used the previous twelve months as a recall period. Third, the cross-sectional design of the survey did not allow any conclusion in terms of a specific causal direction. Fourth, although the total sample size was substantial, the small sample size of some of the categories might have resulted in large uncertainty intervals of their estimated association with an injury incident. Finally, this study did not evaluate interventions on safety issues for health workers, which remain to be investigated in future studies. It can however be safely argued that the nature of this study as the first assessment of NSSIs among a considerable number of health care workers at two tertiary hospitals in Mongolia far outweighs these disadvantages.

In conclusion, NSSIs are common risk factors for infection among health care workers at two of the largest public tertiary hospitals in Ulaanbaatar, Mongolia. For the effective prevention of these injuries, health policy makers and hospital administrators should formulate strategies to improve the working conditions of health care workers, discourage excessive use of injections, and increase their adherence to universal precautions. Reducing the risk of NSSIs through strengthened occupational health standards and safety management systems would eventually decrease the burden of disease on society from infections with blood-borne pathogens in Mongolia.

## Competing interests

The authors declare that they have no competing interests.

## Authors' contributions

MK, NI, and KS conducted the statistical analysis, interpreted the data, drafted the manuscript, and critically revised the manuscript for intellectual content. MA designed the study, collected the data, and critically revised the manuscript. BE and MT designed the study, supported the data collection, and critically revised the manuscript. CK designed the study, supported the data collection, critically revised the manuscript, obtained funding, and supervised the study. All authors read and approved the final manuscript.
